# Classifying Self-Reported Rheumatoid Arthritis Flares Using Daily Patient-Generated Data From a Smartphone App: Exploratory Analysis Applying Machine Learning Approaches

**DOI:** 10.2196/50679

**Published:** 2024-05-14

**Authors:** Julie Gandrup, David A Selby, William G Dixon

**Affiliations:** 1 Centre for Epidemiology Versus Arthritis University of Manchester Manchester United Kingdom; 2 Department of Computer Science Technische Universität Kaiserslautern Kaiserslautern Germany; 3 Department of Rheumatology Northern Care Alliance NHS Foundation Trust Salford United Kingdom

**Keywords:** rheumatoid arthritis, flare, patient-generated health data, smartphone, mobile health, machine learning, arthritis, rheumatic, rheumatism, joint, joints, arthritic, musculoskeletal, flares, classify, classification, symptom, symptoms, mobile phone

## Abstract

**Background:**

The ability to predict rheumatoid arthritis (RA) flares between clinic visits based on real-time, longitudinal patient-generated data could potentially allow for timely interventions to avoid disease worsening.

**Objective:**

This exploratory study aims to investigate the feasibility of using machine learning methods to classify self-reported RA flares based on a small data set of daily symptom data collected on a smartphone app.

**Methods:**

Daily symptoms and weekly flares reported on the Remote Monitoring of Rheumatoid Arthritis (REMORA) smartphone app from 20 patients with RA over 3 months were used. Predictors were several summary features of the daily symptom scores (eg, pain and fatigue) collected in the week leading up to the flare question. We fitted 3 binary classifiers: logistic regression with and without elastic net regularization, a random forest, and naive Bayes. Performance was evaluated according to the area under the curve (AUC) of the receiver operating characteristic curve. For the best-performing model, we considered sensitivity and specificity for different thresholds in order to illustrate different ways in which the predictive model could behave in a clinical setting.

**Results:**

The data comprised an average of 60.6 daily reports and 10.5 weekly reports per participant. Participants reported a median of 2 (IQR 0.75-4.25) flares each over a median follow-up time of 81 (IQR 79-82) days. AUCs were broadly similar between models, but logistic regression with elastic net regularization had the highest AUC of 0.82. At a cutoff requiring specificity to be 0.80, the corresponding sensitivity to detect flares was 0.60 for this model. The positive predictive value (PPV) in this population was 53%, and the negative predictive value (NPV) was 85%. Given the prevalence of flares, the best PPV achieved meant only around 2 of every 3 positive predictions were correct (PPV 0.65). By prioritizing a higher NPV, the model correctly predicted over 9 in every 10 non-flare weeks, but the accuracy of predicted flares fell to only 1 in 2 being correct (NPV and PPV of 0.92 and 0.51, respectively).

**Conclusions:**

Predicting self-reported flares based on daily symptom scorings in the preceding week using machine learning methods was feasible. The observed predictive accuracy might improve as we obtain more data, and these exploratory results need to be validated in an external cohort. In the future, analysis of frequently collected patient-generated data may allow us to predict flares before they unfold, opening opportunities for just-in-time adaptative interventions. Depending on the nature and implication of an intervention, different cutoff values for an intervention decision need to be considered, as well as the level of predictive certainty required.

## Introduction

Rheumatoid arthritis (RA) is characterized by fluctuations in disease severity over time, with periods of worsening referred to as “flares.” Flares represent a significant burden on patients, including uncontrollable symptoms and compromised ability to perform everyday tasks [1], and are associated with negative outcomes such as loss of functional ability and structural damage [2,3]. To minimize the impact of significant flares on the patient, a flare must be identified early, so that necessary interventions can be initiated. However, changes in disease severity often occur between scheduled visits to a clinician (usually every 6-12 months) which might hamper optimal disease management. In the early stages of a flare, patients self-manage and then progress to seeking medical help when they feel they are losing control [4]. Understanding when a flare is happening—or about to happen—could remove some of the barriers to seeking help.

Patient-generated health data, including patient-reported symptoms, could play an increasingly important role in clinical decision-making [5]. Smartphones, tablets, and wearable devices can facilitate the collection of self-reported symptom data between scheduled clinical appointments and at a much higher frequency, for example, daily or weekly. This would allow us to “listen in” on the short-term patterns of RA disease severity and identify flares earlier or even predict flares before they unfold. The ability to identify or predict flares between clinical appointments based on patient-generated data would potentially allow for timely interventions. These might include self-management advice, medication adjustment, triggering a remote consultation, or bringing forward a planned visit. Just-in-time adaptive interventions are an emerging area of research that, until now, has primarily been deployed in mental health and behavior-change treatments [6,7]. Before using predictive algorithms, however, it is important to understand how well the prediction performs and whether such performance would be acceptable in a clinical setting.

Due to the potentially high-dimensional and nonlinear nature of intensively collected patient-generated data, modern machine learning (ML) methods could offer benefits over traditional tools, such as logistic regression, for accurate prediction. ML is increasingly being used in rheumatology, for example, Hügle et al [8]. However, the literature on predicting distant outcomes, such as flares through longitudinal patient-generated health data, is still in its infancy and currently limited by heterogeneity in predictors, flare definitions, frequency of data collection, and classification methods [9,10].

In previous work, we investigated the association between patient-reported flares and daily symptom scores [11]. The purpose of this analysis was to build on this work by investigating the feasibility of using ML methods to classify self-reported RA flares based on a small data set of daily symptom data collected through a smartphone app. Specifically, the objectives of this exploratory study were (1) to fit 3 binary classifiers and consider their performance, (2) to illustrate the initial implications of different cutoff values for predicting a flare, and (3) to frame an agenda for future work supporting ways to meaningfully leverage digital patient-generated health data to predict flares and improve patient outcomes.

## Methods

### Data

This study was a post hoc analysis of data from the first phase of the Remote Monitoring of Rheumatoid Arthritis (REMORA) study [12], which involved 20 patients with RA using a smartphone app to track their daily symptoms over 3 months.

Participants received prompts every evening to report several symptoms on a 0-10 numerical rating scale based on the RAID scale adapted for daily use [13]: pain, function (“difficulty in doing daily activities”), fatigue (attributed to RA), sleep quality, overall physical and emotional well-being, and ability to cope. Users reported the duration of morning stiffness daily using 1 of 7 time intervals. Weekly questionnaires asked patients about self-assessed tender and swollen joint counts and the binary flare question: “Have you experienced a flare in the last week?” These questions were prompted by a notification every 7 days to complete the weekly question set. Eligibility criteria were (1) clinician-verified RA, (2) treated at a specific outpatient clinic, (3) willingness to participate, and (4) able to provide written consent. The app and its content were co-designed with patients, clinicians, and researchers. For further details of the REMORA study, see Austin et al [12].

### Definition of Outcomes and Explanatory Variables

We treated each weekly flare report as a binary outcome. It was left up to the patient to decide what was classified as a flare. Weeks with an unanswered (missing) flare question are not included in this analysis.

To fit a binary classification model, it was necessary to extract a “feature vector” or list of predictors from the sequence of daily symptom data that were mapped to each weekly flare report. The 7 days up to and including each flare report were treated as the exposure period. For each exposure period, the following 5 symptom summary features were calculated for each of the 8 daily symptoms: minimum, maximum, mean score, SD, and slope. Isolated daily reports (those not followed by a flare report in the next 6 days) were discarded, so every remaining exposure period contained at least 2 daily data points. Although not prompted, participants were able to answer the weekly flare question at any time during the week outside of the 7-day schedule, resulting in some partially overlapping exposure periods. In that case, we allowed the intersecting daily symptom reports to correspond to multiple outcomes. Where the same participant responded more than once on the same date, we assumed later-recorded responses superseded earlier ones.

The patient-reported symptom scores were collected using integer numerical rating scales from 0 to 10 (morning stiffness on a 7-point ordinal scale). For this exploratory analysis, all symptoms were treated as continuous variables. This approach was chosen because it allows ease of comparison with other work in intraindividual pain variability [14]. Additionally, distributions of pain scores, both during “flare” and “no-flare” weeks, were not noticeably skewed, suggesting minimal influence of ceiling or floor effects. One alternative to our approach would be incorporating monotonic constraints in the ML model, however, there were no readily available packages for monotonic random forests in either Python or R at the time of writing. However, the imposition of monotonic constraints, while improving the interpretability of the model, is as likely as not to decrease predictive performance.

### Statistical Analysis

ML classification concerns the task of recognizing objects and being able to separate them into categories. With our analysis, we aimed to classify each week as either a flare week or a non-flare week based on the symptom summary features. While the most popular binary classification models are simpler ones, like logistic regression, the seemingly high‐dimensional and nonlinear nature of disease activity motivates more complex “black box” ML approaches including random forest classifiers. We fitted 3 distinct classes of binary classification models to the data: logistic regression with and without elastic net regularization, a random forest, and naive Bayes. Random forest models use decision trees as building blocks. Decision trees use features to divide the observations into subgroups (or classes) that are as different from each other as possible. Many decision trees operate as an ensemble and the class selected by most trees will become the final output. Under a naive Bayes classifier, continuous predictors may be assumed to follow independent univariate normal distributions with a separate mean and variance estimated for each class. Given a feature vector (list of predictor values), the predicted class is then inferred probabilistically via the Bayes rule [15]. These 3 methods were chosen based on previous work that aimed to predict flares in RA [16,17] and in addition, they have the benefit of being straightforward to fit on a generic binary classification problem as presented here.

When evaluating the performance of classifiers, a training data set is required for fitting them and another distinct data set is needed for the subsequent evaluation and test of those classifiers. We trained our models using the R package mlr3 [18]. Fine-tuning of the models beyond the default settings was not performed for this exploratory study and no imputation was performed on missing values. A 10-fold cross-validation was performed, with 18 (90%) participants comprising the training sets and the remaining 2 (10%) participants comprising the test sets. The validation was repeated 10 times, each time reserving 2 different participants for testing. In the case of longitudinal data collected from individuals, the training-test data splits should fall between participants, so that data associated with a particular patient fall entirely in a training set or a test set, so testing and training are not performed within the same patient timeline. In other words, the models were tested on different patients to those on which they were trained [16]. We then evaluated the performance of each of the models against patient-reported flares as the gold standard according to the area under the curve (AUC) of the receiver operating characteristics curve. The model with the highest AUC in the test data set was considered the best final model.

We considered sensitivity and specificity for 10 different thresholds in order to illustrate different ways in which the predictive model could behave in a clinical setting. Sensitivity is the proportion of those with a flare who have a positive prediction, while specificity is the proportion of those without a flare that has correctly been predicted to have no flare. We did this by setting the sensitivity from 0.5 to 0.9 in 0.1 unit increments, and then doing the same for specificity (Table 1). Corresponding positive predictive values (PPVs), that is, the probability that those with a predicted flare indeed go on to have a flare, and negative predictive values (NPVs), that is, the probability that those with a predicted non-flare indeed do not experience a flare, were also considered for these different thresholds to illustrate their potential impact and clinical utility.

**Table 1:** Sensitivity, specificity, positive and negative predictive values, and implications at different cutoffs. Shown for logistic regression with elastic net regularization.

Cutoffs	Psychometric properties			
	Sensitivity	Specificity	Positive predictive value	Negative predictive value
Cutoff 1	0.50	0.90	0.65	0.83
Cutoff 2	0.60	0.80	0.53	0.85
Cutoff 3	0.70	0.74	0.49	0.87
Cutoff 4	0.80	0.72	0.51	0.90
Cutoff 5	0.90	0.43	0.37	0.92
Cutoff 6	0.88	0.50	0.39	0.92
Cutoff 7	0.87	0.60	0.44	0.92
Cutoff 8	0.83	0.70	0.51	0.92
Cutoff 9	0.60	0.80	0.53	0.85
Cutoff 10	0.50	0.90	0.65	0.83

### Ethical Considerations

The original study was reviewed and approved by the Greater Manchester Central Research Ethics Committee (15/NW/0172). All participants completed informed consent forms which included consent for secondary use of deidentified data for research purposes. Participants did not receive any financial compensation for their participation.

## Results

**Figure 1:**
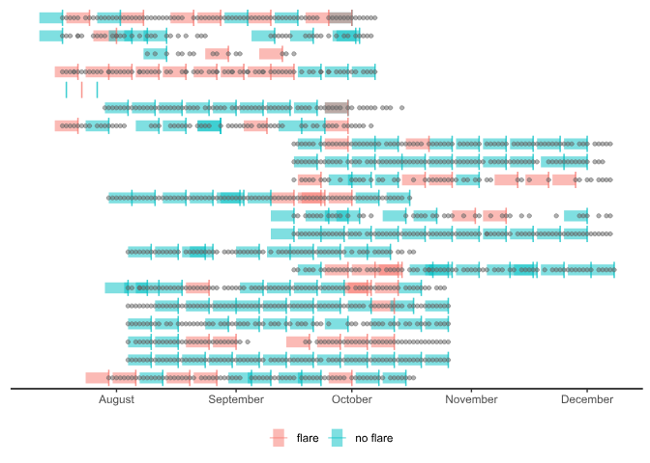
Patterns of daily and weekly data entry. Each row is a different participant. Vertical lines denote weekly responses and points denote daily responses. The shaded bands represent the week preceding each weekly response and the two colors denote whether patients reported a flare or no flare in that week.

The collected data set comprised 20 unique participants completing a total of 1325 daily and 213 weekly questionnaires. Each participant reported an average of 61 daily reports and 11 weekly reports over a median follow-up time of 81 (IQR 79-82) days. Of the participants, 60% (n=12) were female, all except 1 were White British, and the mean age was 57 (SD 11) years. Patterns of daily and weekly responses for each app user are shown in Figure 1.

Participants reported a median of 2 (IQR 0.75-4.25) flares each throughout the study resulting in 57 flares in total. The largest number of flares reported by a single participant was 9, while 5 participants reported no flares at all.

**Figure 2:**
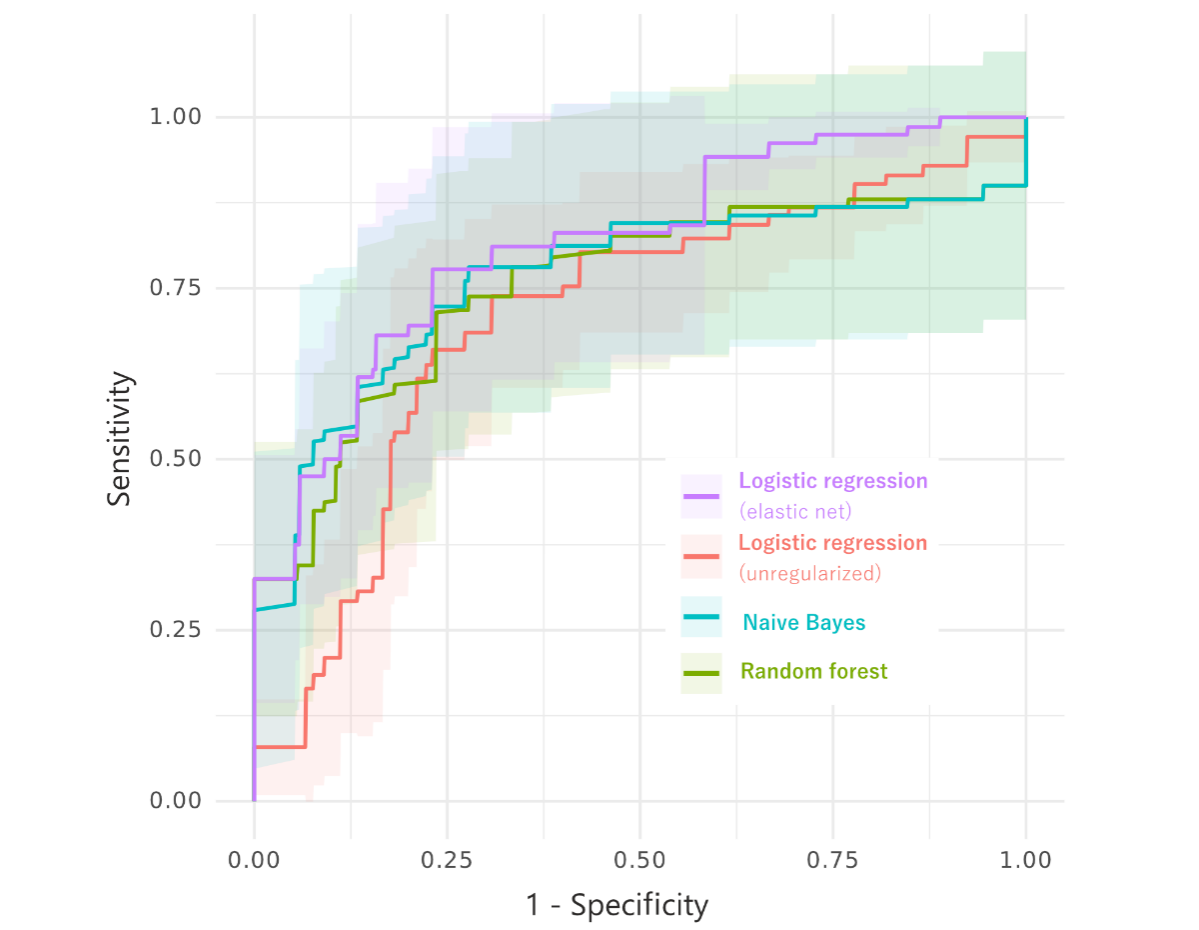
Classifier performance for each of the 4 models.

Classifier performances are visualized in Figure 2. AUCs were broadly similar for all models, but the model with the highest AUC was the logistic regression with elastic net regularization with an AUC of 0.82. This was followed by naive Bayes and random forest with AUCs of 0.77 and 0.75, respectively. Unregularized logistic regression, as expected, had the lowest AUC of 0.71. Figure S1 in Multimedia Appendix 1 shows precision-recall curves, and Figure S2 in Multimedia Appendix 1 shows the average relative importance of each predictor for the random forest model and logistic regression (data not shown).

Table 1 shows sensitivity, specificity, PPVs, and NPVs for a range of different thresholds for the model with the highest AUC. At a cutoff requiring specificity to be 0.80, the corresponding sensitivity to detect flares was 0.60 for the regularized logistic regression model, meaning that the prediction model correctly identified 3 in every 5 self-reported flares, and correctly identified 4 in every 5 non-flares. At this cutoff, and given the prevalence of flares within our data set, the PPV was 0.53 and the NPV was 0.85, meaning there was (only) a 53% chance that the patient actually had a flare after the algorithm predicted a flare, but an 85% chance the patient did not have a flare, if the algorithm predicted a non-flare.

For that same model, we also considered a different threshold that favored identifying true positives, that is, the ability to correctly identify those reporting a flare. At a cutoff requiring sensitivity to be 0.80, the corresponding specificity was 0.72. The PPV was 0.51 and the NPV was 0.90 for this threshold. Of all the sensitivity and specificity options, ranging from 0.5 to 0.9, the greatest PPV was 0.65 (with an associated NPV of 0.83) and the highest NPV was 0.92 (where the best corresponding PPV was 0.51).

## Discussion

### Principal Findings

With this exploratory study, we showed that it is feasible to use robust ML methods to classify patient-reported flares based on daily symptom scorings in the preceding week with decent accuracy. Of the 3 classifiers fitted, logistic regression with elastic net regularization had the highest overall AUC of 0.82, but across the different models, AUCs were broadly similar. Random forest classifiers tend to overfit, especially for high-dimensional data. Therefore, any advantage of random forest for our data set is most likely due to overfitting on the small number of observations, which might explain the seemingly better performance of the regularized logistic regression model. For the model with the highest AUC, at a cut point requiring specificity to be 0.80, sensitivity to detect flare was 0.60, resulting in the accurate prediction of 3 out of 5 flares from the prior week’s daily symptom data. Given the prevalence of flares in this cohort, the best PPV we could achieve meant only around 2 of every 3 positive predictions were correct (PPV 0.65). If we instead prioritized a higher NPV, we could correctly predict over 9 in every 10 non-flare weeks, although this meant the accuracy of predicted flare weeks fell to only 1 in 2 being correct (NPV and PPV 0.92 and 0.51, respectively). In the future, it will be necessary to find the optimal balance between identifying true flares (or, in other words, not missing flares) without overburdening the health service by identifying flares incorrectly. Models were fitted to a relatively small data set of 20 highly selected patients with RA with 3 months of daily symptoms, so interpretations should be cautious. Nonetheless, our study serves as an early indicative example of how the classification of flares based on daily patient-generated data is ambitious but feasible.

Other examples of predicting RA flares using longitudinal patient-generated health data (in contrast to using longitudinal clinical routine data) are sparse. Haynes et al [9] attempted to classify weekly‐reported flares from a combination of daily RA symptom scorings and weekly flare questionnaires collected on a smartphone. Similar to our results, their best-performing logistic regression classification model had an AUC of 0.81 and, at a cutoff requiring specificity to be ≥0.80, sensitivity to detect flare was 0.62 [9]. As an alternative to patients actively entering the data, Gossec et al [10] predicted weekly patient-reported flares based on passively collected step counts from fitness trackers in 155 patients with RA and axial spondyloarthritis. Using a naive Bayes classification model, they found that patient-reported flares were strongly associated with physical activity and proposed that processing of patient-level physical activity data using ML can be used to accurately detect flares [10]. Similarly, Rao et al [19] demonstrated the ability of physical activity tracker data to classify health status over time (not specifically flares) in patients with RA. Creagh et al [20] observed that augmenting standard patient-reported outcomes with objective sensor-based data improved the estimation of RA severity levels. Combined, these results raise the possibility for passive surveillance that might, in the future, lead to just-in-time adaptive interventions without the need for continuous active symptom tracking.

### Limitations

The methodology of our study has several limitations. First, as already mentioned, the data set is limited in size, which makes the interpretation of results more challenging and additionally limits the possibility of meaningful interpretation of the importance of different predictors for classifying a flare. Additionally, most patients were of White British ethnicity, which limits the generalizability of our results to other populations. Second, laboratory data, such as c-reactive protein, erythrocyte sedimentation rate, or traditional disease activity measures, were not available. This means we cannot correlate patient-reported data with clinician-reported information and limits our understanding of the generalizability of the population (that said, it would not be plausible to have a clinician assessment whenever a patient experiences a flare). We fitted several different models, but the lack of an external validation data set also limits the generalizability of our results. There is a need to externally validate our findings in a larger, more diverse data set. Third, for modeling purposes, we treated the original ordinal features as continuous. This preserves the information in the ordering but requires the assumption that the numerical distance between each category is approximately equal. We assumed that this was reasonable for our analysis, but other more complex methods could be used to account for ordinal data [21]. Fourth, the feature vectors also do not account for temporal dependence (or autocorrelation) within or between patient weeks, that is, the fact that pain today may depend on pain yesterday, or that the likelihood of reporting a flare this week is affected by reports in previous weeks. Fifth, isolated daily scores—those not within 7 days of a subsequent flare report—were discarded. However, in a different analysis approach, these could be treated as censored observations. We included variables from the week prior to the self-reported flare and not any data from preceding weeks. We, therefore, did not assess how far in advance it was possible to predict a flare: we would want better performance from the more proximal data before extending the time window further. Sixth, the period of symptom tracking was limited to 3 months. While we know patients can sustain symptom tracking for up to 6 months [22], we do not yet know how much longer they would continue, nor whether the predictive algorithm is stable over time. Finally, our definition of flare was a nonvalidated, pragmatic, patient-centered one, which left it to the patient to decide when a flare occurred, and therefore it could be interpreted differently by different patients. Multiple definitions of RA flares have been suggested [23,24], but to date, no reference standard has been agreed upon. This might consequently make it harder to predict a “flare” if each patient’s interpretation of a flare is different. There is a need to develop a validated, accepted, and easy-to-use (digital) flare definition in RA which can be used prospectively.

While the sensitivity and specificity of a test are stable, PPVs and NPVs are influenced by the prevalence of the disease in the population. When prevalence decreases, the PPV decreases too. In contrast, the NPV will increase. The prevalence of patient-reported flares in our cohort therefore influences predictive values, and its broader usability is dependent on our cohort’s representativeness of the broader RA population.

### Clinical Implications

Our results point to a future where real-time analysis of frequently collected patient-generated data from symptom tracking may allow us to predict imminent flares before they unfold. This in turn opens opportunities for just-in-time adaptive interventions. Just-in-time adaptive interventions “leverage mobile technology to deliver the right type of support, at the right time based on ongoing information about the individual’s internal state and context” [25]. Until now, they have primarily been deployed in supporting health behavior change [7,26], but they hold enormous potential for fluctuating diseases, like RA, where timely intervention for an increase in disease activity is beneficial. Depending on the nature and implication of a just-in-time adaptive intervention, different cutoff values for an intervention decision need to be considered. Because of cost and other implications, different interventions will require different levels of predictive certainty before an action is triggered. In RA, we could imagine, say, 2 different scenarios in response to a predicted flare: One where self-management advice is newly offered or promoted via a notification within the app, and a second where a scheduled clinical consultation is brought forward based on the data entered by the patient. Striking the right balance between missing true flares and flagging up false positives is crucial. We might tolerate serving up automated written self-management advice for more false positives because the implications are relatively few. This would also mean we rarely miss the opportunity to provide advice to someone with a true flare that the predictive test has failed to identify. On the other hand, we need more caution when offering a clinical consultation. Here, tolerance for false positives should be low because of the high implications—scheduling an expensive consultation in an already busy and overworked clinic because a flare is predicted, but where that consultation is wasted as there is no true flare. In this instance, a high PPV of the algorithm is essential. If we apply these considerations to our results, we could foresee that self-management could be usefully delivered in response to the predicted flares. Whether incorrectly promoting self-management advice to 1 in every 2 people who might have a flare (PPV=0.51 and NPV=0.92) would need formal evaluation to see if this is indeed acceptable. Conversely, given the current model performance and prevalence of flares, we would be unlikely to use the predictive model to trigger a time- and resource-intensive clinical intervention because, at best, only 2 in 3 of these predicted flares would be correct (PPV=0.65 and NPV=0.83). In addition to careful assessment of quality, cost-benefit analyses of various interventions depending on model performance will be a key piece to the assessment of predictive models in clinical practice. For example, evaluating whether the costs of additional telephone consultations or clinic visits in response to automated flare predictions are outweighed by the benefits of earlier interventions including potential shorter recovery, earlier return to work or quality of life, and better long-term outcomes.

### Conclusions

Classifying self-reported flares based on daily symptom scorings in the preceding week using ML methods was feasible in this exploratory study, with regularized logistic regression seeming to outperform the other ML methods in this small data set. The observed predictive accuracy may improve as we obtain more data and external validation in larger data sets is an important next step. As we begin to understand how we can use regular symptom tracking data to predict imminent flares in RA before they unfold, we in turn open opportunities for just-in-time adaptive interventions. This is now a tangible future, but more data and more research are needed to realize the goal of using ML to offer a personalized care approach that ultimately improves patient outcomes.

## Appendix

Multimedia Appendix 1 Supplementary figures.

## Abbreviations

AUC: area under the curve
ML: machine learning
NPV: negative predictive value
PPV: positive predictive value
RA: rheumatoid arthritis
REMORA: Remote Monitoring of Rheumatoid Arthritis
ROC: receiver operating characteristics

## Data Availability

The data sets analyzed during this study are not publicly available due to containing information that could compromise research participant consent but are available from the corresponding author on reasonable request
