# Supplementary material for: Classifying Self-Reported Rheumatoid Arthritis Flares Using Daily Patient-Generated Data From a Smartphone App: Exploratory Analysis Applying Machine Learning Approaches
Source: JMIR Form Res. 2024 May 14;8:e50679. doi: 10.2196/50679 (PMC11134244; doi:10.2196/50679)

**Supplementary Figure S1.** Precision-recall curves

**
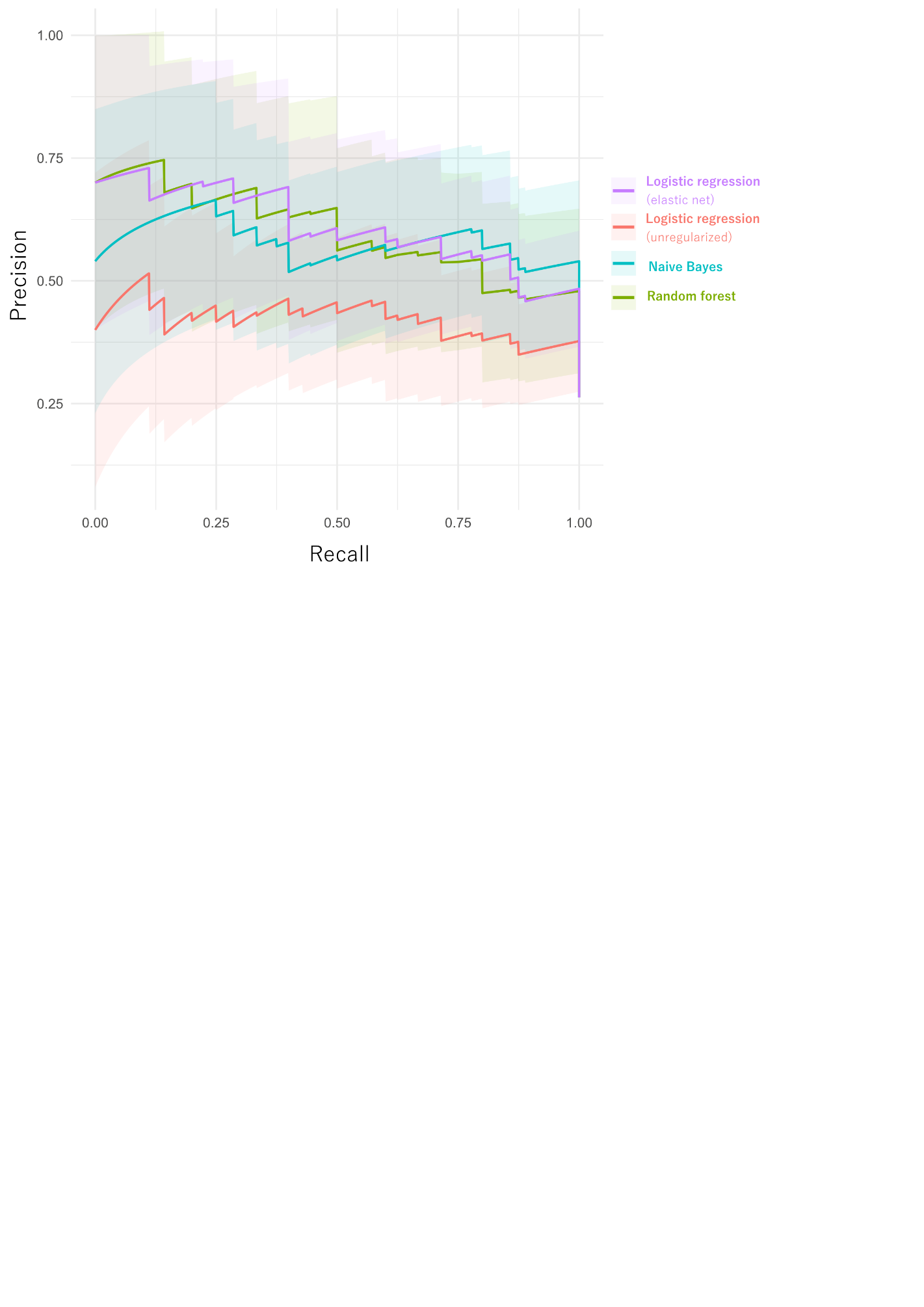
**

**Supplementary Figure S2**. Estimated importance of predictors in a) logistic regression model and b) random forest classifier, averaged over folds of cross-validation. Error bars represent 2 standard errors from the mean.

**a**


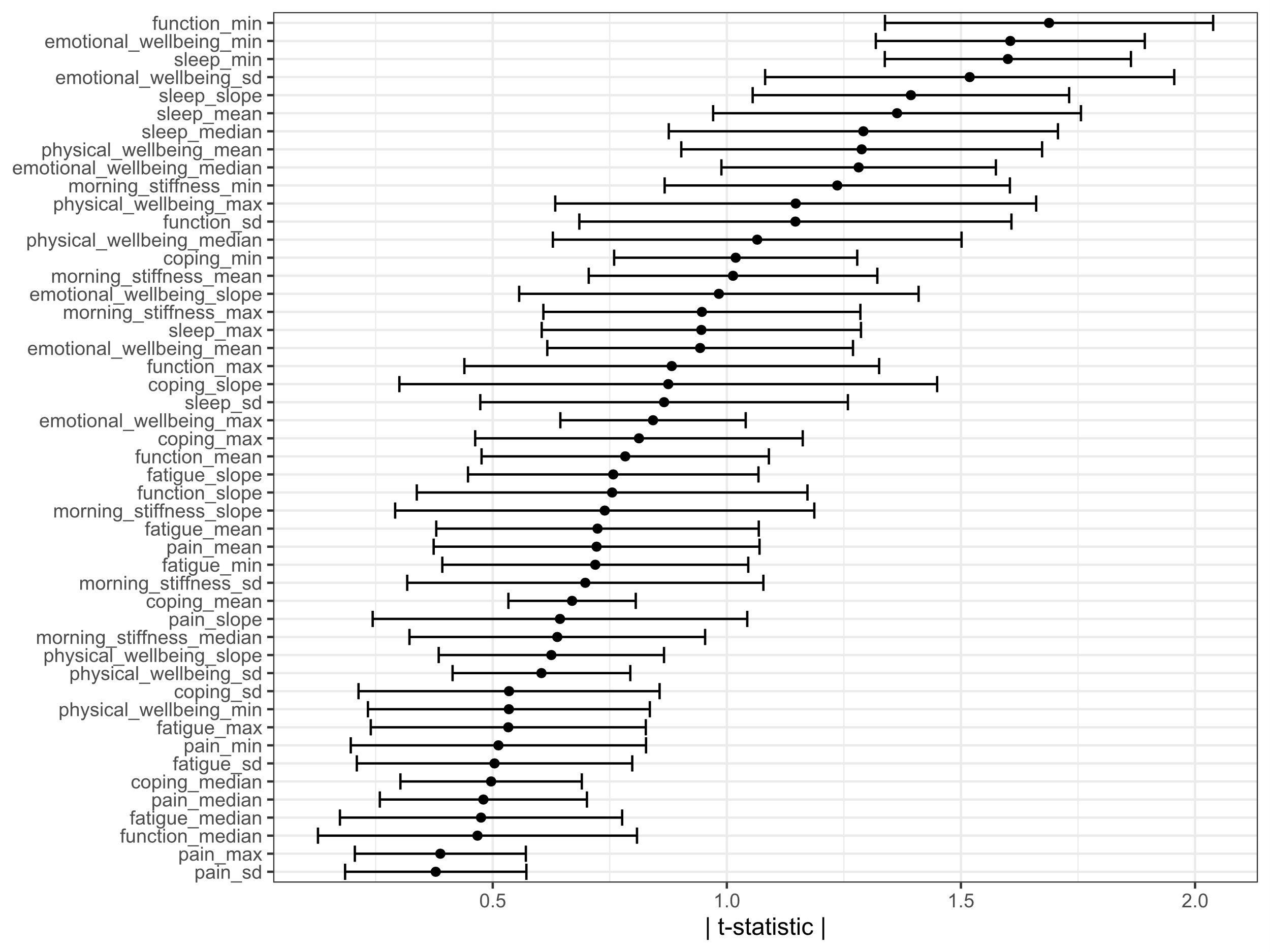


**b**


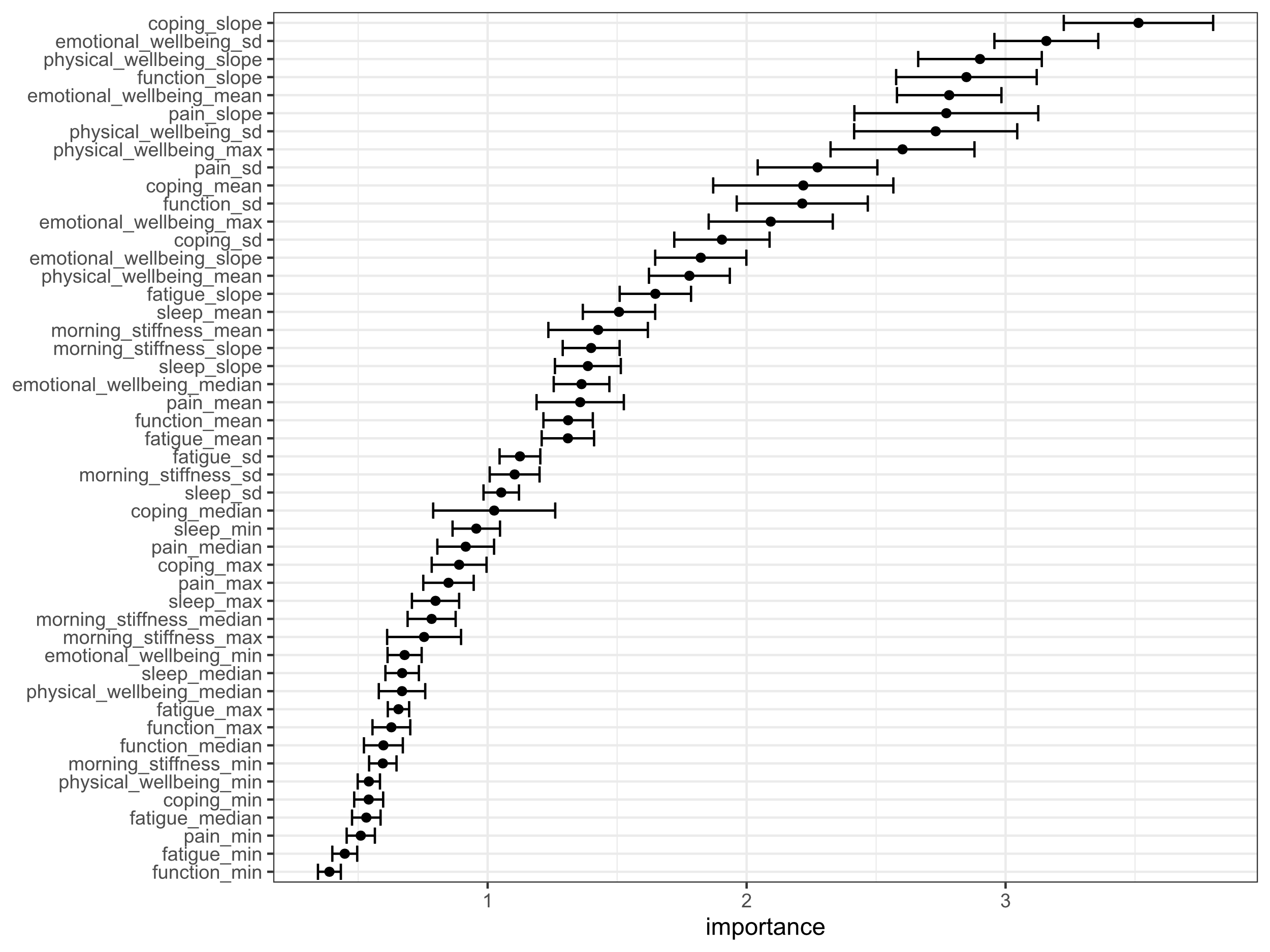

Supplement: Multimedia Appendix 1 [file formative_v8i1e50679_app1.docx]
